# MicroRNAs in Alzheimer’s Disease: Diagnostic Markers or Therapeutic Agents?

**DOI:** 10.3389/fphar.2019.00665

**Published:** 2019-06-18

**Authors:** Francesco Angelucci, Katerina Cechova, Martin Valis, Kamil Kuca, Bing Zhang, Jakub Hort

**Affiliations:** ^1^Memory Clinic, Department of Neurology, 2nd Faculty of Medicine, Charles University and Motol University Hospital, Prague, Czechia; ^2^International Clinical Research Centre, St. Anne’s University Hospital, Brno, Czechia; ^3^Department of Neurology, University Hospital Hradec Králové, Charles University in Prague, Faculty of Medicine in Hradec Králové, Hradec Králové, Czechia; ^4^Department of Chemistry, Faculty of Science, University of Hradec Kralove, Hradec Králové, Czechia; ^5^Department of Radiology, Affiliated Drum Tower Hospital of Nanjing University Medical School, Nanjing, China

**Keywords:** microRNAs, Alzheimer’s disease, biomarker, diagnosis, therapy

## Abstract

MicroRNAs (miRNAs) are small non-coding nucleic acids able to post-transcriptionally regulate gene expression by binding to complementary sequences of target messenger RNA (mRNA). It has been estimated that at least 1% of the human genome encodes miRNA and every miRNA can regulate up to 200 mRNAs. These findings suggest that dysregulation of miRNA expression could be associated with several human pathological conditions including central neurological disorders. Alzheimer’s disease (AD) is a neurodegenerative disorder and the most common cause of dementia in the elderly. The characteristic symptoms are a progressive loss of memory and other cognitive functions due to the impairment of particular types of neurons and synapses, leading to neuronal death. At present, the available symptomatic treatments can only slow down disease progression without stopping it. miRNAs are widely found within the nervous system where they are key regulators of functions such as neurite outgrowth, dendritic spine morphology, neuronal differentiation, and synaptic plasticity. This has been the clue for considering miRNAs crucial molecules to be studied in AD, and nowadays, dysfunction of miRNAs in AD is increasingly recognized. In this review, we summarized existing evidence about miRNAs as biomarkers or therapeutic agents. The field of miRNAs as biomarkers is more advanced in terms of human data, and it is likely that miRNAs will be used successfully in the near future. Given the huge number of miRNAs potentially involved in diagnostics, miRNA panels will be used for specific tasks such as the stage of the disease, the risk prediction, and disease progression. The field of miRNAs as therapeutics is rapidly developing, and it offers a huge variety of solutions. These include positive effects related to beta-amyloid or tau reduction, increased number of neurons, inhibition of apoptosis, protection of synapses, transformation of other cellular elements into missing/deficient neurons in AD, and so on. It is predictable that both areas of research will be carried forward. However, given the absence of an AD therapy able to stop or reverse the disease, it is desirable to accelerate research on miRNAs as therapeutic agents.

## MicroRNAs

MicroRNAs (miRNAs) are small non-coding nucleic acids (around 20 nucleotides) that are able to post-transcriptionally regulate gene expression by binding to complementary sequences of target messenger RNA (mRNA) (Friedman et al., 2008). The effect of miRNA on genes is due to their binding to the 3′ untranslated region (3′UTR) of the target mRNAs, which leads to translational inhibition of the target gene or mRNA degradation (Czech and Hannon, 2010). Since the discovery of the first miRNA in 1993 (Lee et al., 1993), it has been estimated that at least 1% of the human genome encodes miRNA and every miRNA can regulate up to 200 mRNAs (Chen et al., 2012). These findings have suggested the idea that the dysregulation of miRNA expression could be associated with several human pathological conditions (Bartel, 2004; Lekka and Hall, 2018). Indeed, the amount of data on the involvement of miRNA in human diseases is enormous and includes serious diseases such as cancer (Zhang et al., 2018b), cardiovascular disorders (Wojciechowska et al., 2017), and peripheral (Zhang et al., 2018a) as well as central (Deverman et al., 2018) neurological disorders.

Besides that, the scientific interest in miRNAs has mainly focused on two areas of investigation: 1) miRNAs as possible disease biomarkers, and 2) miRNAs as a molecular target of disease therapies. In this review, we will focus the attention on one of the most devastating central nervous system (CNS) disorders, Alzheimer’s disease (AD), and will try to summarize the evidence for a role of miRNAs as diagnostic markers and/or therapeutic agents.

## MicroRNAs’ Involvement in Alzheimer’s Disease

AD is a complex neurodegenerative disorder and the most common cause of dementia in the elderly (Jagust, 2018). The characteristic symptoms are a slow but progressive loss of memory and other cognitive functions due to the impairment of particular types of neurons and synapses, finally leading to neuronal death. Pathological processes are caused by impaired metabolism of beta-amyloid (Aβ) followed by tau pathology (Hunter et al., 2018). Recently, there has been a shift in scientific interest from fibrillar amyloid-forming plaques to shorter species, i.e., monomeric and oligomeric forms of Aβ. Aβ is an amino acid fragment, composed by 40 or 42 amino acids, derived from the proteolytic cleavage of a longer precursor, called amyloid precursor protein (APP) (Roher et al., 2017). Although these features of the disease are very well documented (Jeong, 2017), unfortunately, the treatments aimed at reducing production or removing already produced misfolded proteins are still not a successful strategy (Coimbra et al., 2018). At present, the available symptomatic treatments can only slow down disease progression, without stopping it (Adlimoghaddam et al., 2018).

An alternative approach has focused on genetics as many genes encoding for proteins of the CNS have been proposed as candidates to explain AD pathogenesis. Many of them encode for proteins involved in synaptic regulation and plasticity, or for these misfolded Aβ and tau proteins (Carmona et al., 2018). In particular, miRNAs are widely found within the nervous system where they are key regulators of functions such as neurite outgrowth, dendritic spine morphology, neuronal differentiation, and synaptic plasticity (Vreugdenhil and Berezikov, 2010;Cao et al., 2016). This has been the clue for considering miRNAs crucial molecules to be studied in AD, and nowadays, dysfunction of miRNAs in AD is increasingly recognized (Reddy et al., 2017b).

Many miRNAs have been identified as key elements for the regulation of cognitive functions and memory processes lost in AD, through the regulation of activity-mediated protein synthesis at the synaptic level (Ramakrishna and Muddashetty, 2019). Mice overexpressing the miR-34c are characterized by reduced dendritic length and spine density (Kao et al., 2018). In an AD mouse model (Tg2576 mice), it was found that miR-124 is dramatically increased in the hippocampus and is directly associated with deficits in synaptic plasticity and memory dysfunction (Wang et al., 2018). These data suggest that memory deficits in AD may be caused by miRNA alterations. Nonetheless, it should be noted that miRNA expression may in turn be influenced by epigenetic mechanisms that lead to effects on cognition (Harman and Martín, 2019). It has been shown that the beneficial effects of physical exercise on cognition can be mediated by changes in the expression of certain miRNAs (Fernandes et al., 2017). Interestingly, these effects can also occur in neuropathological conditions. Mice exposed to traumatic brain injury showed recovery of hippocampus-related cognitive deficits associated with modulation of miRNAs (miR-21 and mir-34) after spontaneous running wheel (Bao et al., 2014). As for AD, in a spontaneous senescence-accelerated P8 mouse model (SAMP8), it was shown that spontaneous running wheel modulates the expression of several miRNAs involved in AD, such as miR-30a-5p and miR-128 (Cosín-Tomás et al., 2014).

Several studies have used profiling strategies to identify miRNA dysregulation in AD. In cellular and animal models of AD, there are miRNAs that have consistently been identified as dysregulated. In the brains of transgenic animals harboring the human presenilin 1 (PS1) and a Swedish APP mutant (APP_Swe_/PS1 mice), as an *in vitro* AD model, a reduction of miR-298 and miR-328 was found, which was associated to higher β-amyloid precursor protein converting enzyme (BACE1) protein (Boissonneault et al., 2008). In SAMP8 mice, the level of miR-195 was also negatively related to the protein level of BACE1 (Zhu et al., 2012). In mouse PC12 cells and primary cultured hippocampal neurons exposed to toxic concentration of Aβ, it was found that miR-124 also negatively correlates with BACE1 expression (Makeyev et al., 2007;Fang et al., 2012). In APP_Swe_/PS1 mice, overexpression of miR-98 was found to induce Aβ production and phosphorylation of tau, whereas inhibition of miR-98 reduced them (Hu et al., 2013).

Many other miRNAs have also been recently identified, and their number is continuously increasing (Fransquet and Ryan, 2018). Some of them have been linked to altered regulation of key genes known to be involved also with AD (Millan, 2017). The direction of miRNA changes in AD could be different. Some of them are upregulated while others are repressed (Chen et al., 2018). Accordingly, either a protective or an inducing role for different miRNAs in AD has been postulated (Reddy et al., 2017a).

Despite this, the importance of miRNAs in AD is still a matter of debate. The main reasons lie in the fact that the results of many studies are not reproducible and there is the possibility that the studied miRNAs may undergo variations for causes different from those elicited by AD.

Thus, the data on miRNAs in *in vitro* and *in vivo* AD animal models must be confirmed by studies in the human brain. This aspect is crucial for establishing the real involvement of miRNAs in AD. Unfortunately, these data are not yet numerous.

In primary cultures of human brain and in brain specimens from AD patients, it has been shown that some miRNAs are in fact altered and may have effects on Aβ deposition (Long et al., 2012; Long et al., 2014;Jiang et al., 2018) or be in turn deregulated by pro-inflammatory transcription factors (Zhao et al., 2014).

MiR-137 inhibits p-tau overexpression induced by Aβ1–42 in human SH-SY5Y neuroblastoma cells (Jiang et al., 2018). In addition, brain levels of miR-137 measured by quantitative reverse transcription polymerace chain reaction (RT-qPCR) in the hippocampus, cerebral cortex, and serum of APP/PS1 mice were found reduced (Jiang et al., 2018). MiR-153 was analyzed by RT-qPCR (hsa-miR-153; ID:001191) in frozen brain specimens of the frontal cortex (from the Harvard Brain Tissue Resource Center) in age-matched control and AD patients (Long et al., 2012). MiR-153 was reduced in the specimens of AD brains. In addition, miR-153 delivery in primary human fetal brain cultures significantly reduced APP expression, while antisense miR-153 inhibitor significantly elevated APP expression.

In short postmortem interval brain tissue-derived extracellular fluid from AD and age-matched control neocortex, carefully selected from multiple domestic and international brain bank sources, a consistent upregulation of several brain-enriched miRNAs that are under transcriptional control by the pro-inflammatory transcription factor NF-kB (Zhao et al., 2014) was observed (Alexandrov et al., 2012). MiRNA were measured by miRNA array analysis using a human MRA-1001 miRNA microfluidic chip analytical platform. Upregulated miRNAs include miRNA-9, miRNA-34a, miRNA-125b, miRNA-146a, and miRNA-155 (Alexandrov et al., 2012). It should, however, be noted that, also in postmortem studies, RNA quality obtained from human samples usually displays a high range of variability (Clement et al., 2016). These methodological aspects are only becoming addressed in the most recent years (Duda et al., 2018).

## MicroRNAs as Diagnostic Markers in Alzheimer’s Disease

The fact that AD patients are characterized by miRNA alterations in the brain and in biological fluids, including serum, plasma, and cerebrospinal fluid (CSF), has prompted the idea to use these noncoding sequences as biomarkers of the disease (Zendjabil, 2018). At present, the only biomarkers currently recognized for AD are Aβ peptides and tubulin-associated unit (tau) proteins (Hampel et al., 2018). These measurements in CSF and blood have, however, some limitations. Besides the issues related to the standardization of the methodology in body fluids, there is not enough evidence for adopting cutoff values of these biomarkers (alone or in combination) in the current clinical practice (Ritchie et al., 2013). In addition, predictive value of these biomarkers in patients with mild cognitive impairment (MCI) is still matter of debate (Paolacci et al., 2017).

Prediction in asymptomatic individuals to develop symptomatic AD (MCI or dementia) is important (Shaffer et al., 2013). Furthermore, there is currently no therapeutic agent capable of curing or preventing AD. Thus, biomarkers can be used to evaluate the efficiency of therapeutic agents under testing in clinical trials and so accelerate the therapeutic discovery process.

MiRNAs, unlike mRNAs, are stable enough in biological fluids, including serum, plasma, and CSF (Zhang et al., 2018c). In addition, many of them target genes directly involved in AD pathophysiology such as presenilins, BACE-1, APP (Liu et al., 2014), TOMM40 (Wang et al., 2019), and BDNF (Croce et al., 2013; Keifer et al., 2015). Moreover, there is evidence that, in APOE4 mice, the association between certain miRNAs (miR-146a) and inflammatory mediators (NFκB) seems to be even greater (Lusardi et al., 2016; Teter et al., 2016).

Analysis of miRNAs in body fluids is a relatively simple procedure (Kalogianni et al., 2017) and a non-invasive approach. If we compare miRNAs to conventional protein-based biomarkers of AD, the level of sensitivity achieved for miRNAs due to amplification by PCR is far superior to what is currently available for proteins (Kumar et al., 2017). In addition, the cost of miRNA quantification is far lower than that of established biomarkers, such as structural magnetic resonance imaging (MRI) and molecular neuroimaging with positron emission tomography (PET).

For this purpose, many miRNAs appear to be promising. In a study with postmortem brain tissue and serum from AD patients, MCI, and controls, it was found that miR-455-3p was upregulated in the AD group. The miRNA quantification was performed with Affymetrix microarray analysis and validated using RT-qPCR (Kumar and Reddy, 2018). In addition, the levels of miR-455-3p and miR-34a-5p measured in plasma from a homogeneous AD cohort positively correlated with Aβ1–42 levels in CSF (Cosín-Tomás et al., 2016). In another study, it was found that miR-93 expression measured by RT-qPCR was markedly decreased in AD patients’ serum compared with controls (Kiko et al., 2014).

In a set composed of 36 clinically diagnosed AD patients and 22 age-matched cognitively normal controls, it was found that serum miR-501-3p levels (RT-qPCR) were downregulated in AD patients, and its lower levels significantly correlated with lower Mini-Mental State Examination scores (Hara et al., 2017). The research is still ongoing and many other miRNAs seem to be useful as AD biomarkers (Fransquet and Ryan, 2018).

One limitation could be the inconsistency in miRNA profiling data in different studies. Most of the human data on miRNAs and AD derive from studies in serum, plasma, and CSF. The quantity of variables in these studies is enormous and includes the nature of the biological liquid (serum/plasma vs. CSF), the measurement methods (like microarray and RNA sequencing), and the algorithms necessary to associate the miRNAs with a certain state of the disease. This has been evidenced in a recent meta-analysis (Takousis et al., 2017) that screened data for more than 1,000 miRNAs in AD and concluded that the most significant findings were observed with miR-342-3p in blood and miR-127-3p in CSF.

In very recent reviews (Martinez and Peplow, 2019; Swarbrick et al., 2019), the need to have very standardized parameters has been highlighted. The most important parameters include recruitment of patients, gender, inclusion and exclusion criteria, medications, most appropriate validation methods, normalization, and statistical analysis of data.

Numerous strategies have been proposed for normalization for miRNA profiling methods (Kumar and Reddy, 2016). It should be noted that this problem is not specific to AD, but rather general concerns of the use of circulating miRNAs as biomarkers in blood to reflect brain diseases. Furthermore, due to their great number and different action on genes, it is unlikely that a single miRNA measurement will be adopted as biomarker.

In a recent study (Denk et al., 2018), it is highlighted how the use of cluster analysis of a miRNA family, rather than the study of a single miRNA, seems to be a better strategy to find potential AD biomarkers. Thus, each panel will include a set of miRNAs for specific tasks, such as synaptic function, neuronal survival, AD disease staging, AD specificity versus other diseases causing dementia, and so on (Zafari et al., 2015;Guo et al., 2017).

Regarding the choice of the optimal biological fluid to be used for analysis, a recent study showed that the use of serum, instead of CSF, provides appreciable results, as the data on miRNA correlate satisfactorily with the state of cognitive functions and with the changes in cortical integrity (Maldonado-Lasuncion et al., 2018). It is hoped that future studies will continue to address these aspects so that a sensitive, specific, and minimally invasive test could be developed.

## MicroRNAs and Treatment of Alzheimer’s Disease

The use of miRNAs to treat human diseases is rapidly developing. In 2018, the FDA approved the first therapy based on administration of miRNA for the treatment of rare progressive polyneuropathy caused by hereditary transthyretin-mediated (hATTR) amyloidosis (Adams et al., 2018; Wood, 2018).

The observation that miRNAs interfere with (or modulate) expression of candidate genes in AD has suggested the researchers to develop miRNA-based therapeutic strategies. This is also because the therapies targeting Aβ have not been successful in treating or even slowing down the disease (Alifragis and Marsh, 2018). Depending on the data available, a mimic or an antagonist of miRNAs could be explored as therapeutic agent. The possibility to modulate mRNA expression of an AD candidate gene has been recently explored by using antisense oligonucleotides (ASO), which act similarly to miRNA by inhibiting mRNA transcription (DeVos et al., 2017). Interestingly, it was shown that the injection of ASO into the CSF of nonhuman primates causes the reduction of the target RNA (tau) in the brain regions analyzed, including the hippocampus (DeVos et al., 2017). The relevance of this study was the demonstration that injection of ASO into CSF may modulate brain mRNA expression. Based on these data, as reported at the 70th annual meeting of the American Academy of Neurology, a clinical trial to test a tau ASO in people with mild AD has been started by Biogen, IONIS Pharmaceuticals (https://www.alzforum.org/therapeutics/biib080).

The therapeutic modulation of miRNAs can be done in two ways. In the first case, the miRNA function is inhibited through a single-stranded complementary antisense oligonucleotide (ASO). In the second case, the expression of the miRNA is increased or brought back to its physiological level through the administration of compounds that stimulate its production or through a double-stranded synthetic oligonucleotide miRNA that has the same functions as endogenous miRNA (miRNA mimic).

The most recent studies on AD animal models and cell cultures demonstrated that restoring the expression of certain miRNAs could have positive effects that include neuroprotection, recovery of cognitive function, and neuronal regeneration. It has been shown that an increase in miRNA expression may counteract Aβ and tau accumulation in AD cellular and animal models by acting on different molecular pathways.

Using miRNA microarray analysis of cortical tissue from Tg2576 transgenic mice, miRNAs of the miR-200 family (miR-200b and miR-200c) were identified as downregulators of Aβ secretion by modulation of mTOR in murine primary neurons and human neuroblastoma cells (Higaki et al., 2018). A similar effect on downregulation of Aβ production was observed after upregulation of miR-330 in murine AD model through the activation of MAPK signaling pathway (Zhou et al., 2018). In the SH-SY5Y cell line transfected with APP_Swe_, it was found that miR-15b reduces Aβ by inhibiting the expression of the enzyme BACE1 (Li and Wang, 2018). Similarly, in a study aimed to explore the role of miR-124 in the pathogenesis of AD, miR-124 expression was examined using RT-qPCR analysis in 35 cases of sporadic AD brain tissues and 35 cases of normal control subjects (An et al., 2017). MiR-124 expression was significantly reduced in AD brain tissues compared with the control group (An et al., 2017). In addition, inhibition of miR-124 significantly increased BACE1 levels in human neuroblastoma (SH-SY5Y) cells while miR-124 overexpression significantly suppressed BACE1 expression (An et al., 2017). In brain tissue taken at autopsy from patients with AD and from those with severe primary age-related tauopathy, it was found that miR-219 measured by RT-qPCR analysis is downregulated (Santa-Maria et al., 2015). In addition, in the *Drosophila* model (that produces human tau), it was demonstrated that reduction of miR-219 exacerbates tau toxicity, while overexpression of miR-219 partially abrogates this effect (Santa-Maria et al., 2015).

Neuronal regeneration can be achieved by overexpressing other miRNAs. In AD animal models, it was shown that miR-302/367 convert astrocytes to neurons that replace dead ones (Ghasemi-Kasman et al., 2018) while miR-9 stimulates the differentiation of neuronal stem cells into neurons (Li et al., 2017).

Recovery of cognitive functions can occur by upregulation of miRNAs acting at the synaptic level. Upregulation of members of the miR-188 family was found to be effective in enhancing synaptic activity and, importantly, restoring cognitive functions in AD mouse models (Zhang et al., 2014;Lee et al., 2016). Instead, miR-214 (Zhang et al., 2016) and miR-let-7f-5p (Han et al., 2018) may positively act on cognitive deficits by inhibition of autophagy, leading to increased viability and decreased caspase-mediated apoptosis.

In contrast, overexpression of other classes of miRNAs has negative effects on neurons, and they therefore need to be downregulated. In cell cultures, it has been observed that inhibition of miR-128 decreases Aβ-mediated cytotoxicity through inactivation of the NF-κB pathway (Geng et al., 2018). In addition, miR-125b (Ma et al., 2017) and miR-146a (Wang et al., 2016) overexpression promotes neuronal apoptosis and tau phosphorylation in AD cellular models. Moreover, other negative effects on neuronal survival elicited by miRNAs include the downregulation of neurotrophic factor expression, as demonstrated in human neuroblastoma cells (Croce et al., 2013) and AD transgenic mice (Lee et al., 2012).

These are only a few of the most recent examples of direct miRNA applications as a therapeutic approach. The action of many other miRNAs was investigated and comprehensive reviews on the matter already exist (Di Meco and Praticò, 2016; Gupta et al., 2017).

The pharmacological modulation of miRNAs can be achieved by the use of many compounds. For example, it was shown that anti-inflammatory drugs could be of benefit in preventing the progression of AD *via* modulation of miRNA expression (Shadfar et al., 2015;Huang et al., 2017). In addition, natural compounds known for their potential as neuroprotective agents in AD, such as resveratrol (Khou et al., 2017) and osthole, seem to exert their action by modulating specific miRNAs and activating processes such as autophagy and neuronal regeneration (Li et al., 2017). Furthermore, recent data support the hypothesis that exosomes, small vesicles secreted by neurons and glial cells, may serve as therapeutic agents to deliver miRNAs and/or short interfering RNA (siRNA) in AD patients (Chen et al., 2017).

As evinced, the field of intervention of miRNAs as therapeutic agents in AD is very wide and it is likely to be further extended in the next years by the use of computerized gene analyses (Pang et al., 2017). Nonetheless, possible limitations to clinical application come from delivery difficulties and the specificity of miRNA silencing. In fact, each single miRNA may have very pleiotropic effects to the genome, and thus alteration of miRNA expression in humans may lead to undesired side effects in AD patients (Junn and Mouradian, 2012).

## Conclusions and Future Directions

In this review, we summarized existing evidence about miRNAs as biomarkers or as therapeutic agents (**Table 1**). These two fields of investigation are constantly evolving; thus, the amount of data available is enormous and difficult to synthesize. The current evidences suggest that miRNAs may serve as both biomarkers and therapeutic agents in AD.

**Table 1:** Most recent MiRNAs associated with AD pathophysiological hallmarks.

Direction of changes	MiRNA (known target)	Reference	Pathologic process	Biomarker	Therapeutic target
	miR-342-3p	Takousis et al., 2017	Aβ accumulation	X	
	miR-455-3p	Kumar and Reddy, 2018		X	
	miR-146a (NF-κB pathway)	Kiko et al., 2014; Wang et al., 2016	Aβ accumulation Inflammation	X	X
**Upregulated**	miR-34a	Cosín-Tomás et al., 2016	Cell death	X	
	miR-30a-5p (BDNF)	Croce et al., 2013	Synaptic dysfunction		X
	miR-206 (BDNF)	Lee et al., 2012			X
	miR-128 (NF-κB pathway)	Geng et al., 2018	Tau toxicity		X
	miR-106b	Huang et al., 2017			X
	miR-330 (MAPK pathway)	Zhou et al., 2018			X
	miR-195 (BACE1)	Zhu et al., 2012	Aβ accumulation		X
	miR-200 (mTOR)	Higaki et al., 2018			X
	miR-9	Li et al., 2017	Aβ accumulation Inflammation		X
**Downregulated**	miR-15 (BACE1)	Li and Wang, 2018	Aβ accumulation Tau toxicity Cell death		X
	miR-93	Dong et al., 2015		X	
	miR-127-3p	Takousis et al., 2017	Cell death	X	
	miR-214	Zhang et al., 2016			X
	miR-let-7f-5p	Han et al., 2018			X
	miR-124 (BACE1)	An et al., 2017	Synaptic dysfunction		X
	miR-188	Lee et al., 2016; Zhang et al., 2014			X
	miR-219	Santa-Maria et al., 2015	Tau toxicity		X

Alterations of several miRNAs are present in Alzheimer’s disease and may be responsible for its pathophysiological hallmarks by altering the expression of the genes involved in the pathologic process. AD, Alzheimer’s disease; BDNF, brain-derived neurotrophic factor; NF-κB, nuclear factor kappa-light-chain-enhancer of activated B cells; BACE1, Beta-secretase 1; mTOR, mammalian target of rapamycin.

The field of miRNAs as biomarkers is more advanced in terms of human data, and it is likely that miRNAs will be used successfully as biomarkers in the near future. However, as previously mentioned, it is more probable that, given the huge number of miRNAs potentially involved in diagnostics, miRNA panels will be used for specific functions. These functions include the stage of the disease, the risk prediction of conversion from MCI to AD, and disease progression.

The field instead of miRNAs as therapeutics is rapidly developing, and it offers a huge variety of solutions. These include positive effects related to Aβ or tau reduction (Li and Wang, 2018), increased number of neurons (Geng et al., 2018), inhibition of apoptosis (Zhang et al., 2017), protection of synapses (Lee et al., 2016), transformation of other cellular elements into missing/deficient neurons in AD (Ghasemi-Kasman et al., 2018), and so on.

In the near future, it is predictable that both areas of research will be carried forward. However, given the current absence of an AD therapy able to stop, if not reverse, the disease, it is desirable to accelerate research on miRNAs as therapeutic agents.

## Author Contributions

FA drafted the manuscript. KC and JH performed critical editing. MV, KK, and BZ participated in constructive outline, discussions, and editing. All authors read and approved the final manuscript.

## Funding

This work was supported by project no. LQ1605 from the National Program of Sustainability II (MEYS CR), the European Regional Development Fund—Project ENOCH (No. CZ.02.1.01/0.0/0.0/16_019/0000868), the Institutional Support of Excellence 2 LF UK (Grant no. 699012), and the Grant Agency of Charles University (Project no. 176317).

The study was also supported in part by the grants from the Ministry of Education, Youth and Sports of the Czech Republic (PROGRES Q40) and from the Ministry of Health of the Czech Republic (RVO–FN HK 00179906). This work was also supported by University of Hradec Kralove (Faculty of Science, VT2019-2021) and by the National Natural Science Foundation of China (81720108022, BZ).

## Conflict of Interest Statement

The authors declare that the research was conducted in the absence of any commercial or financial relationships that could be construed as a potential conflict of interest.
